# Production and characterization of bacterial cellulose by *Rhizobium* sp. isolated from bean root

**DOI:** 10.1038/s41598-024-61619-w

**Published:** 2024-05-13

**Authors:** Raed A. H. Almihyawi, Elshan Musazade, Nazeer Alhussany, Sitong Zhang, Huan Chen

**Affiliations:** 1 College of Life Sciences, Jilin Agricultural University, Changchun, 130118 China; 2 Department of Quality Control, Baghdad Water Authority, Baghdad, 10011 Iraq; 3 College of Life Sciences, Sana University, Sana, 1247 Yemen; 4 Key Laboratory of Straw Biology and Utilization, Ministry of Education, Changchun, 130118 China

**Keywords:** Bacterial cellulose, *Rhizobium* sp., *Komagataeibacter hansenii*, Media optimization, Nisin, Antibacterial activity, Biochemistry, Biological techniques, Biotechnology, Microbiology

## Abstract

Bacterial cellulose (BC) is a natural polymer renowned for its unique physicochemical and mechanical attributes, including notable water-holding capacity, crystallinity, and a pristine fiber network structure. While BC has broad applications spanning agriculture, industry, and medicine, its industrial utilization is hindered by production costs and yield limitations. In this study, *Rhizobium* sp. was isolated from bean roots and systematically assessed for BC synthesis under optimal conditions, with a comparative analysis against BC produced by *Komagataeibacter hansenii*. The study revealed that *Rhizobium* sp. exhibited optimal BC synthesis when supplied with a 1.5% glucose carbon source and a 0.15% yeast extract nitrogen source. Under static conditions at 30 °C and pH 6.5, the most favorable conditions for growth and BC production (2.5 g/L) were identified. Modifications were introduced using nisin to enhance BC properties, and the resulting BC-nisin composites were comprehensively characterized through various techniques, including FE-SEM, FTIR, porosity, swelling, filtration, and antibacterial activity assessments. The results demonstrated that BC produced by *Rhizobium* sp. displayed properties comparable to *K. hansenii*-produced BC. Furthermore, the BC-nisin composites exhibited remarkable inhibitory activity against *Escherichia coli* and *Pseudomonas aeruginosa*. This study contributes valuable insights into BC’s production, modification, and characterization utilizing *Rhizobium* sp., highlighting the exceptional properties that render it efficacious across diverse applications.

## Introduction

Cellulose, a water-insoluble polysaccharide, is derived from various organisms, including animals, plants, algae, bacteria, and cell-free systems^1,2^. It stands out as the most prevalent renewable biopolymer within the biosphere, positioning it as a favorable candidate for the escalating need for biocompatible and environmentally sustainable applications. However, the rising demand for cellulose products derived from plants has led to heightened wood consumption, consequently exacerbating deforestation–a significant worldwide environmental concern^3^. Despite plant cellulose's unparalleled abundance as the most prevalent natural polymer globally, with an annual production of 1.5 × 10^12^ tons, its multifaceted nature has resulted in its utilization across diverse sectors, including the food and textile industries. However, impurities such as lignin, hemicellulose, and pectin limit their applicability in cosmetic and medical industries^4–6^. Bacterial cellulose (BC), also known as microbial cellulose, is synthesized by various bacterial species, such as *Komagataeibacter*, *Agrobacterium*, *Rhizobium*, *Azotobacter*, *Achromobacter*, *Aerobacter*, *Pseudomonas*, *Sarcina*, *Alcaligenes*, and *Salmonella*^7–9^. *Komagataeibacter hansenii*, a Gram-negative acetic acid bacterium, is known to produce considerable amounts of BC and has been extensively studied as a model microorganism for BC production^10,11^.

Plant cellulose and BC share similar chemical compositions, yet the self-assembled nanofiber structure of BC distinguishes it from plant cellulose^7,12^. Possessing an ultrafine network structure, BC showcases distinctive physicochemical properties, including (1) an ultrafine nanofibrous network structure measuring 50–120 nm, (2) elevated purity, (3) a high degree of polymerization, (4) pronounced crystallinity, (5) considerable tensile strength, (6) remarkable water absorption, and retention capabilities, and (7) inherent biocompatibility. These excellent properties make this material a promising candidate for a wide range of value-added applications, including biomimetic sensory actuators, wearable electronics, functional 3D carbon-based nanomaterials, flexible displays, soft tactile devices, artificial muscles, artificial vessels, wound dressings for skin injury repair, 3D scaffolds for tissue engineering, and carriers for drug delivery. BC has numerous biomedical applications, such as artificial blood vessels in microsurgery, polymer scaffolds for cartilage and bone regeneration, membranes for transdermal drug delivery, and materials for wound dressings in skin burn repair^13–16^. Studies have shown that BC can be loaded with small molecules, such as octenidine, diclofenac, and lidocaine, macromolecules, such as peptides, and polymeric drugs, such as laccase, albumin, and lipase. BC-based drug delivery systems are used in various fields, including transdermal patches, wound dressings, and dental and oral care^17^.

In searching for novel and highly effective BC producers, researchers have attempted to improve BC production by investigating different nitrogen and carbon sources and various additives^18–21^. The essential factors for BC production include the composition of the fermentation media, which contains nitrogen, carbon, and mineral sources, and various operating conditions such as temperature, pH, dissolved oxygen content, age of the inoculum, and inoculation ratio^22–24^. Exploring alternative nitrogen and carbon sources is pivotal in formulating cost-effective media to attain optimal BC yields for large-scale industrial applications. While Hestrin-Schramm (HS) media stand as the prevailing choice for BC production, their expense and the requirement for supplemental items like yeast, glucose, and peptone present notable constraints. Hence, pragmatic evaluations of production costs and scalability are essential for the widespread utilization of BC^8,25^. Recent investigations have concentrated on bacterial strains capable of cellulose production, economical nutrient sources, and supplementary materials to foster the economical production of BC. Researchers have investigated using waste products from industrial and agricultural activities to optimize BC production yields and reduce associated costs^26^. These include sugarcane molasses^27^, dry olive mill residues^28^, brewer’s yeast waste^29^, wood sugar, confectionery processing waste^30^, fruit processing waste^31^, rice bark^32^, lipid fermentation waste, cotton textile waste^33^, konjac powder, and coffee bean hulls^34^. As an illustration, Castro et al.^35^ successfully produced BC microfibrils (2.8 g/L) from pineapple peel and sugarcane juice utilizing a strain of *Gluconacetobacter swingsii* sp. Moosavi-Nasab et al., explored the viability of employing low-grade date syrup to generate BC with the strain *A. xylinum* PTCC 1734, achieving a fermentation media yield of 4.35 g/L. Utilizing sustainable materials for BC production holds the potential to enhance its sustainability by minimizing the environmental impact associated with industrial waste disposal^36^. Furthermore, the versatility of BC extends beyond its current applications, particularly in large-scale production from low-cost feedstocks, offering prospects for applications in packaging, specialized textiles, and advanced functional materials^37^.

In addition to low-cost BC products, BC with different properties must be modified for various applications. In particular, BC, when used as a scaffold material in tissue engineering, is often enriched with various growth factors to stimulate or regulate the differentiation of specific tissues. BC, with improved mechanical properties and a more porous structure, is the optimal choice for this purpose^38^. Porosity is an essential parameter in blood vessel replacement and neural tissue engineering. Physical/chemical modifications are often required to achieve these properties in vitro and in vivo^39^.

Nevertheless, the efficacy of this modification could be improved due to the active hydroxyl groups in the fibers being engaged in hydrogen bond formation. Modifications targeting hydroxyl groups might interfere with hydrogen bonds, consequently affecting the mechanical properties of the BC membrane^39^. As a result, an alternative approach to meet this requirement is to utilize natural BC membranes with more robust mechanical properties and higher porosity.

Nisin, an antimicrobial peptide derived from Lactococcus lactis with a molecular weight of 3.4 kDa, is approved for use in a variety of applications in several countries. It is favored for its compatibility with both food and pharmaceutical products. It does not disrupt the normal gut microbiota, is non-toxic, preserves the appearance and taste of food, has thermal stability, and effectively inhibits the growth of many pathogenic microorganisms^40^. The ability of nisin to bind to cytoplasmic membranes is a significant contributor to its potent antibacterial activity against spore-forming Gram-positive bacteria. However, it has limited activity against certain Gram-negative species due to restricted access to the internal membrane^41^.

Identifying bacterial strains capable of synthesizing this valuable biotechnological product in large quantities is crucial for expanding BC production and application. Recent research has focused on identifying new sources of producers that can efficiently synthesize cellulose and determining the optimal conditions, such as nitrogen, carbon, temperature, pH, polymeric additives, starting materials, incubation time, and inoculum concentration to maximize cellulose production^27^. A better understanding of the properties of BC and the relationship between its structure and properties will provide more opportunities to disseminate its scope. This study aimed to explore the potential of the *Rhizobium* sp. strain as a novel BC producer, with an assessment of the impact of culture conditions on BC structure and properties. The yield, structure, and morphology of hydrated BC were compared with those of BC produced by *K. hansenii*, a well-known efficient BC producer. The nanofibers generated in the selected media underwent characterization for various structural and physicochemical properties, including Fourier Transform Infrared spectroscopy (FTIR), Field-Emission Scanning Electron Microscopy (FE-SEM), water release rate (WRR), water-holding capacity (WHC), swelling, porosity, filtration, and antibacterial properties. The BC produced by *Rhizobium* sp. exhibited novel properties suitable for biomedical applications, such as scaffolds for tissue engineering and protein-based drug uptake and release.

Furthermore, nisin was incorporated into the material to enhance the antimicrobial activity of the dehydrated BC composite, rendering it effective against various microorganisms, including Gram-positive and Gram-negative bacteria. In order to gain a deeper understanding of the material's properties, a detailed investigation was conducted into the various structural and physicochemical properties of both BC and BC-nisin composite membranes. Moreover, the in vitro antimicrobial activity of the BC-nisin composite membranes was evaluated.

## Materials and methods

### Materials

Glucose, yeast extract, peptone, ethanol, agar, citric acid, tryptone, fructose, mannitol, NH_4_Cl, (NH_4_)_2_SO_4_, Na_2_HPO_4_ × 12H_2_O, K_2_HPO_4_, MgSO_4_, NaCl, CaCO_3_, and nisin were used to prepare the culture media, and for the analytical tests. All media components were purchased from Oxoid (Hampshire, UK). Antibiotics (penicillin, ampicillin, tetracycline, and chloramphenicol) were purchased from Hangzhou Microbial Reagent Co. Ltd. (Hangzhou, China). All other reagents used were analytical or microbiological grade and available from local companies.

#### Microbial strains

A strain of *K. hansenii,* ATCC 53582, was used for BC production. *Escherichia coli* and *Pseudomonas aeruginosa* from the culture collection of the Department of Biology, College of Science, University of Baghdad, Iraq, were used to determine the antibacterial activity of the composite membrane.

#### Media

A mannitol yeast extract agar (MYA), HS agar media, and their modifications (culture media, broth fermentation media, and production media) were used for BC production. Each medium was tailored to meet specific nutritional requirements and facilitate the desired metabolic activities of the organisms under study. Each medium was prepared according to established protocols and sterilized using appropriate techniques to maintain integrity and prevent contamination.

### Plant material collection statement

Plant material collection adhered to institutional, national, and international regulations. *Vicia faba* is not classified as a protected or endangered species among wild plants in Iraq, hence necessitating no permissions for the research material collection.

### Isolation and characterization of the microorganisms

**Figure 1:**
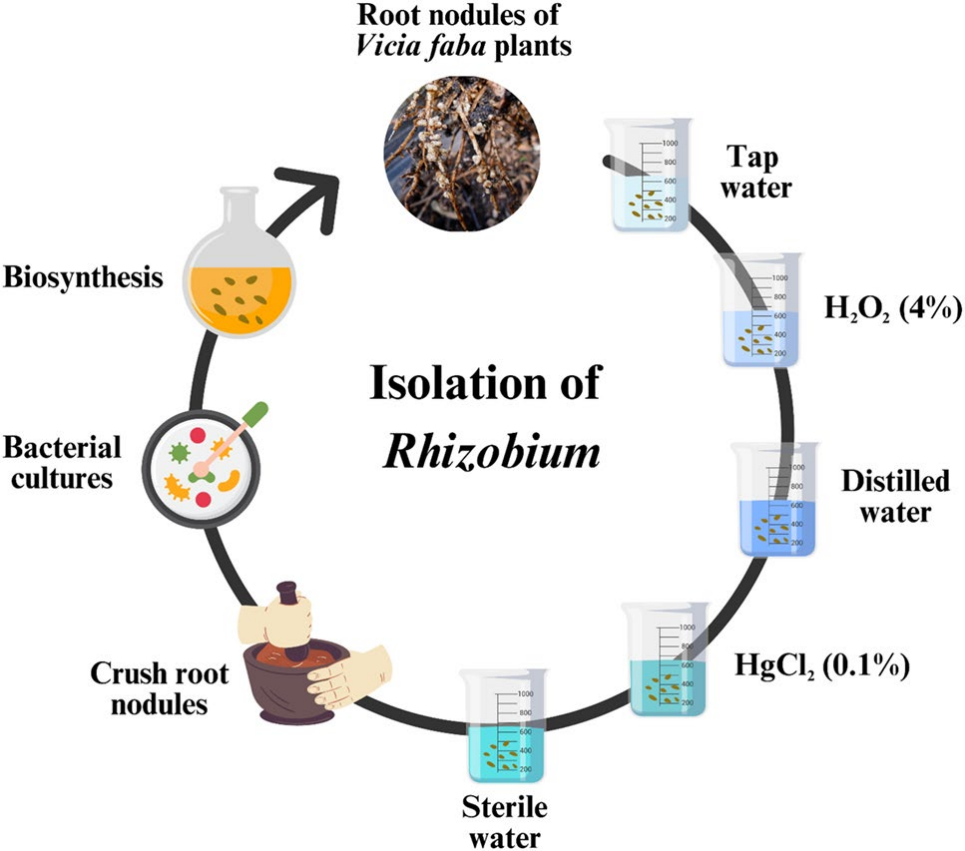
Isolation procedures of *Rhizobium* sp.

The root nodules of *V. faba* plants were gathered from various farms in Aladamya, Baghdad city, Iraq, and placed in a sterile container. Healthy root nodules were carefully chosen, subjected to tap water to eliminate soil adhesion, and cleaned with distilled water. Sterilization was carried out using 95% ethanol and H_2_O_2_ (4%) for a duration of 2 min, followed by maceration and a subsequent wash with sterile water for 3 min. Finally, the nodules underwent immersion in a 0.1% HgCl_2_ solution for 2 min to establish a pure culture of *Rhizobium* sp. (Fig. 1).

#### Morphological identification of Rhizobium sp.

The extracted sample was taken from the suspension and placed on a glass slide for morphological identification and microscopic examination. Their shapes, cell forms, and morphological characteristics were studied using the Gram stain kit. The examination was conducted under a compound light microscope with an oil-immersion lens at 100× magnification. Simultaneously, culture characteristics were assessed by inoculating the suspension on MYA and allowing it to incubate at 25 °C for 3–5 days. The standard culture media consisted of 10 g mannitol, 1 g yeast extract, 0.5 g K_2_HPO_4_, 0.2 g MgSO_4_, 0.1 g NaCl, 0.1 g CaCO_3_, and 15 g/L agar, with an initial pH of 6.8. The resulting solution was transferred to a glass bottle with a 1-L capacity and autoclaved at 121 °C for 15 min^42,43^.

The collected samples were stored in sterile containers at 4 °C and underwent homogenization and serial dilution. Each dilution was spread on HS agar media (20 g/L glucose, 5 g/L yeast extract, 5 g/L peptone, 6.8 g/L Na_2_HPO_4_ × 12H_2_O, 1.5 g/L citric acid, and 20 g/L agar, with an initial pH of 6.0), and incubated for 3 days at 30 °C. After incubation, a single colony was chosen, inoculated into 100 mL/250 mL (100 mL culture media within the 250 mL flask) HS broth media for seed culture, and incubated for 48 h at 30 °C. For BC production, 2% (v/v) of the strained broth was inoculated into 300 mL/500 mL (300 mL culture media within the 500 mL flask) HS broth fermentation media. The microbial cultures were then incubated in treated molasses prepared with 250 mL culture media within the 500 mL flask for 7 days at 30 °C under static conditions. Subsequently, colonies displaying Gram-negative staining and producing white cellulose pellets were selected for further analyses^44^.

#### Biochemical identification of Rhizobium sp.

The biochemical tests for the isolate of *Rhizobium* sp. included various assays performed on bacterial strains isolated from root nodules of the bean under investigation. These tests encompassed the Fluorescence Test^45^, Catalase Test, Voges-Proskuar Test^46^, Gelatin Liquefaction Test^47^, Urease Test^48^, Citrate Utilization Test, Indol Production Test^49^, and Cytochrome Oxidase Test^50^, in addition to assessing bacterial motility, and hydrogen sulfide (H_2_S) gas production test using SIM media^51^.

### Production and purification of BC

The cellulose-producing capabilities of the isolated *Rhizobium* sp. and *K. hansenii* strains were assessed by inoculating flasks with 100 mL of cellulose-producing media and 1 mL of fresh bacterial culture, followed by incubation at 30 °C for one week. The culture media were prepared according to the HS standard: 6.8 g Na_2_HPO_4_ × 12H_2_O, 5 g yeast extract, 1.5 g citric acid monohydrate, 5 g peptone, and finally 20.0 g glucose, all homogenized in 1 L of distilled water. The pH of the solution was adjusted to 6.0. The solution was then transferred to a 1-L glass bottle and autoclaved at 121 °C for 15 min^52^.

Cellulose production was assessed based on the appearance of white cellulose pellicles on the surface of the culture media. Cellulose was extracted from the production media by filtering the cellulose pellicles using Whatman filter paper No. 1, followed by thorough washing with distilled water. The extracted cellulose was decontaminated with a 0.5% NaOH solution at 80 °C for 15 min to eliminate microbial cells and media components. The purified cellulose was then rinsed with distilled water, placed in a Petri dish, and dried in an oven at 105 °C for 1–2 h to determine its dry weight^53^.

### Preparation of the BC composite membrane

The BC membrane was freeze-dried and shaped into circular forms with diameters of 1 cm. Nisin dispersions were prepared in 0.5% (v/v) acetic acid to achieve a final concentration of 1.5% (w/v) and stirred at 37 °C for approximately 3 h. The solution was then oven-dried. The resulting solution was filtered through a polyester cloth to eliminate residual insoluble particles. The BC-dried membrane pellicle was soaked in a nisin-acetic acid solution for 48 h at room temperature to produce a BC-nisin membrane. Various nisin concentrations (100, 200, 300, and 400 IU) were blended to create the BC composite membranes, named cellulose-nisin membranes (BC-Nis).

### Optimization of BC production by *Rhizobium* sp.

#### Determination of the optimal carbon source for BC production

The effect of carbon sources on cellulose production was studied using different substances such as maltose, fructose, and glucose at a concentration of 2% added individually to HS.

#### Determination of the optimal glucose concentration for BC production

The effects of different glucose concentrations were studied, and the best carbon source in the production media was between 0.5 and 3%.

#### Determination of the optimal nitrogen source for BC production

The effect of nitrogen sources on cellulose production was studied using different substances such as yeast extract, peptone, tryptone, NH_4_Cl, and (NH_4_)_2_SO_4_ at a concentration of 0.05% added individually to HS.

#### Determination of the optimal concentration of yeast extracts for BC production

The effects of different concentrations of yeast extract were studied, and the best source of nitrogen in the production media was found to be between 0.05 and 0.2%.

### Environmental conditions

#### pH value

Media with a pH between 5 and 7 were prepared to determine the optimum pH for cellulose production. The pH of the media is adjusted by adding concentrated hydrochloric acid to lower the pH or sodium hydroxide to raise the pH. Simultaneously, the source and concentration of the optimal carbon source and nitrogen concentration for optimal cellulose production were determined based on the results of the above experiments.

#### Temperature

The media were incubated at different temperatures from 25 to 35 °C (25 °C, 30 °C, and 35 °C), with a difference of 5 °C from one media to another, and inoculated with bacterial production. The optimal temperature for cellulose production was determined for 7 days, considering the optimal growth conditions from previous experiments.

#### Aeration

The experiment was conducted in a glass bottle, one filled with air and the other covered with a cotton lid. The following steps prevented contamination of the culture: (1) sterilization of microbiological instruments and media using a convection oven and autoclave; (2) working in a biosafety cabinet; (3) adjusting the incubator temperature to prevent unwanted growth of organisms.

### Characterization of BC

#### Determination of the dry and wet weights of BC

For this purpose, the wet weight of the BC was determined after washing with distilled water. The dry weight was determined using a digital balance (GENIUS, Sartorius, Germany) with an accuracy of 0.001 g after drying^54^.

#### Determination of the thickness of BC membranes

Thickness measurements of the BC membranes were conducted using a digital micrometer (Mitutoyo, no. 293-766; Tokyo, Japan). 10 different points on each BC sample were selected for measurement, and the average values were calculated^55^.

#### Characterization of composite membrane FE-SEM analyzes

To examine the structural properties of the pure freeze-dried BC/K.h, and BC/K.h-Nis membranes, as well as BC/Rhiz and BC/Rhiz-Nis membranes, the BC membranes were fractured using liquid nitrogen, attached to a substrate with carbon tape, and coated with a thin layer of gold. The internal microstructure of the BC membranes, with a focus on the impregnation of nisin nanoparticles into the BC matrix, was observed using FE-SEM (Hitachi S-4800 and EDX-350 (Horiba), Tokyo, Japan) at an accelerating voltage of 10 kV at room temperature.

#### Fourier Transmission Infra-red Spectroscopy (FTIR) analyzes

FTIR analysis of freeze-dried BC/K.h, BC/K.h-Nis, BC/Rhiz and BC/Rhiz-Nis samples were conducted using a BIO-RAD FTS-7PC FTIR spectrophotometer (Bio-Rad, Cambridge, MA). The experimental conditions for the FTIR measurements were set to a spectral range of 400–4000/cm and a resolution of 0.25/cm.

Additionally, FTIR spectra of the pristine freeze-dried BC, BC/K.h, BC/K.h-Nis, BC/Rhiz and BC/Rhiz-Nis scaffold samples were analyzed using a Perkin-Elmer FTIR spectrophotometer (Perkin-Elmer, Hopkinton, Massachusetts, USA). The experimental conditions for these measurements were also set to a spectral range of 400–4000/cm and a resolution of 0.25/cm. The samples were thoroughly mixed with potassium bromide (KBr) pellets (IR grade; Merck, Germany) to facilitate the analysis. The resulting mixture was then used to obtain IR spectra, which were transferred to a computer for further spectral analysis.

#### The water holding capacity (WHC) and water release rate (WRR)

The water-holding capacities of BC/K.h, BC/K.h-Nis, BC/Rhiz and BC/Rhiz-Nis composites were assessed using the sieve shaking method. The dried BC sheets were immersed in distilled water for an appropriate period to allow complete swelling. The plates were carefully removed from the storage containers using tweezers. Subsequently, the samples were placed in a sieve and shaken vigorously twice to eliminate any remaining surface water before weighing. The samples were then air-dried at room temperature, and their weights were recorded at various time intervals (45 h). To ensure complete moisture removal, the samples were dried in an oven at 60 °C for 24 h and then dried at room temperature. The water-holding capacity (WHC) of each sample was determined using the following formula^56^:

$${\mathbf{WHC}} = \frac{{{\mathbf{Mass\, of\, water\, removed\, during\, drying}} \left( {\mathbf{g}} \right)}}{{{\mathbf{Dry\, weight\, of\, cellulose\, sample}} \left( {\mathbf{g}} \right)}}$$

The WRR was determined by measuring the initial weight of the cellulose samples. These samples were stored under ambient conditions for different durations and weighed periodically until a uniform dry weight was obtained^1^.

#### Porosity analyzes

The porosity of BC/K.h, BC/K.h-Nis, BC/Rhiz and BC/Rhiz-Nis composites were determined using the previously developed method. To ascertain the porosity of BC, it underwent a 12-h immersion in distilled water at ambient temperature. The porosity percentage was calculated using the following formula^57^:

$${\mathbf{Porosity}} \left( \% \right) = \frac{{{\mathbf{wet\,}} {\mathbf{weight}} {-} {\mathbf{dry\,}} {\mathbf{weight}}}}{{{\mathbf{wet\,}} {\mathbf{weight}} {-}{\mathbf{weight\,}} {\mathbf{in\,}} {\mathbf{water}}}} \times 100$$

#### Swelling analyzes

Swelling abilities of BC/K.h, BC/K.h-Nis, BC/Rhiz and BC/Rhiz-Nis composites were also investigated. The membranes were cut into 2 × 2 cm square shapes, dried to constant weight, and subsequently subjected to drying until a constant weight was achieved. After this, water elimination occurred, and the membranes were immersed in deionized water at room temperature^58^. The swelling ratio (SR%) was determined through the application of the following equation:

$${\mathbf{Swelling\,}} {\mathbf{ratio}} \left( \% \right) = \frac{{{\mathbf{Wt}} - {\mathbf{Wd}}}}{{{\mathbf{Wd}}}} 100$$

W_t_ represents the weight of the hydrogel in its swollen state, and W_d_ indicates the hydrogel’s weight when in its dried state. Swelling assessments were performed in triplicate. The average results were then calculated according to the method^59^.

#### Filtration analyzes

The filterability of BC/K.h, BC/K.h-Nis, BC/Rhiz and BC/Rhiz-Nis composites were assessed by passing 100 mL of distilled water through samples dried at 70 °C for 4–6 h and determining the diameter. The time taken for the water to filter through each sample was determined as previously reported^60^.

#### Antibacterial activity of the composite membrane

To investigate the antibacterial activity of cellulose obtained from *K. hansenii* and *Rhizobium* sp., samples were treated with different nisin concentrations for 24 h. The zones of inhibition for *E. coli* and *P. aeruginosa*, cultivated on nutrient agar, were determined utilizing the disk diffusion method. BC membranes lacking nisin served as the control specimens. Subsequently, the agar plates were incubated at 30 °C for a duration of 48 h. The antibacterial activity was assessed by measuring the diameter of the inhibition zone surrounding the BC composite membrane, and the results were quantified in arbitrary units (mm).

#### Data analysis

Each measurement for every group was repeated 3 times (N = 3). The data were presented as the mean standard error for both the control and experimental groups. The experimental data were analyzed using IBM SPSS 24.0, and the statistical significance was determined at p ≤ 0.05. The graphs were generated using GraphPad Prism 9.3 (https://www.graphpad-prism.cn/).

## Results and discussion

### Isolation and characterization of *Rhizobium* sp.

*Rhizobium sp.* was isolated from vital root nodules and subjected to a comprehensive identification procedure based on morphological, cultural, and microscopic characteristics. The morphological and cultural characteristics of the bacterial colonies were determined by analyzing their appearance on MYA. The colonies exhibited large, shiny, milky-white raised circular structures with a conspicuous mucoid surface texture. Microscopic analysis revealed that *Rhizobium sp.* exhibited typical Gram-negative rod-shaped bacterial characteristics.

#### Biochemical characterization and identification of Rhizobium sp.

The results of biochemical tests for the isolated *Rhizobium* sp. showed distinctive characteristics (Table 1). *Rhizobium sp.* colonies on MYA agar plates with Congo Red (CR) indicator displayed a light red color. The strain demonstrated the ability to produce oxidase and catalase enzymes but was negative for indole production, Voges-Proskauer, sodium citrate utilization, H_2_S production, urease, and gelatinase enzymes^61,62^.

**Table 1:** Biochemical test results for *Rhizobium* sp.

Test	Result
Congo red	Light red
Fluorescence test	−
Catalase test	+
Voges-Proskuar test	−
Gelatin liquefaction test	−
Urease test	−
Citrate utilization test	−
Indol production test	−
Cytochrome oxidase test	+
H_2_S production test	−

### Production and purification of BC by *Rhizobium* sp.

The ability of *Rhizobium sp.* to produce cellulose was investigated by culturing the isolate in HS media and determining the dry weight of the cellulose produced. The results showed that *Rhizobium* sp. produced cellulose with a dry weight of 2.5 g/L, which was visually manifested as a white pellicle layer on the surface of the HS media.

### Optimization of BC production by *Rhizobium sp.*

#### Determination of the optimal carbon source for BC production

**Figure 2:**
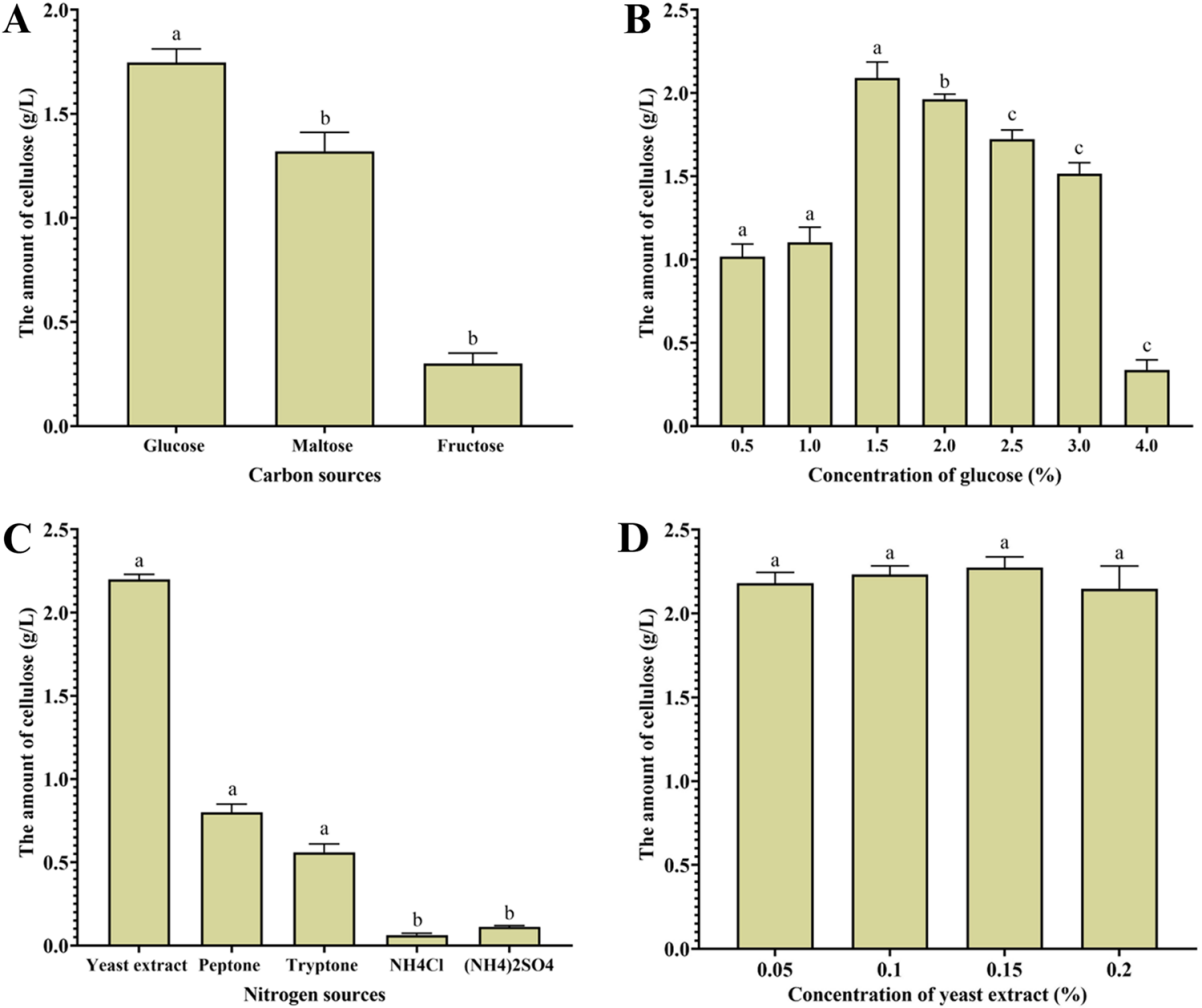
The effect of different concentrations of carbon sources (**A**), glucose (**B**), nitrogen sources (**C**), and yeast extract (**D**) on the production of cellulose in static culture (for nitrogen sources and yeast extract, glucose concentration was 1.5% in static culture). Letters indicate significant differences P < 0.05 when comparing different treatments.

Figure 2A illustrates the effects of different carbon sources on cellulose production by *Rhizobium* sp. The results showed that glucose had the highest cellulose productivity among the sugars. The amount of cellulose produced was 1.75 g/L, followed by maltose at 1.3 g/L and fructose at 0.3 g/L. Consequently, the subsequent experiments chose glucose as the optimal carbon source for cellulose production.

Fructose, a widely available and cost-effective sugar found mainly in industry and waste, has been suggested as the preferred carbon source for cellulose production by certain *Acetobacter* strains, resulting in higher yields. A study has demonstrated the remarkable efficiency of phosphofructokinase in *Acetobacter*, which catalyzes the conversion of fructose-1-phosphate to fructose-1,6-bisphosphate^63^. In another study, *Gluconoacetobacter hansenii* was evaluated for cellulose production using different carbon sources, including glucose, fructose, inulin, and mannitol. The highest cellulose yield was obtained when fructose was used as the sole carbon source^64^.

While it was found that media containing sucrose from subsidized coconut juice was the best media for cellulose production by *A. xylinum*^65^, it was found that higher cellulose production by *A. xylinum* ATCC 10245 bacteria was obtained when glycerol was used as the carbon source. The consumption rates of the carbon sources affected the amount of cellulose product, not only the type^66^. Using both carbon sources, mannitol, and arabitol, resulted in high amounts of cellulose by *A. xylinum* in static cultures^67^.

#### Determination of the optimal glucose concentration for BC production

The effects of different glucose concentrations on cellulose production by *Rhizobium sp.* are shown in Fig. 2B. The addition of glucose to the cellulose production media at a concentration of 1.5% resulted in the production of 2.05 g/L. Cellulose productivity decreased when the glucose concentration increased by 3% and reached 1.52 g/L, whereas a concentration of 4% reached 0.35 g/L. This was probably caused by oxidizing a large amount of glucose, causing it to accumulate in large quantities. The acidity of gluconic media increases due to inhibition of cellulose production. A decrease in the amount of cellulose produced by *K. hansenii* increases the glucose concentration in the production media by 4%^64,68,69^.

#### Determination of the optimal nitrogen source for BC production

The effects of different nitrogen sources on cellulose production by *Rhizobium sp.* are shown in Fig. 2C. Compared to other nitrogen sources, yeast extract showed the highest cellulose productivity. This can be explained by the fact that yeast extract contains significant concentrations of essential vitamins and specific growth hormones. The amount of cellulose produced was 2.2 g/L, followed by peptone at 0.8 g/L and tryptone at 0.56 g/L. Therefore, yeast extract was chosen as the nitrogen source for cellulose production in subsequent experiments because NH_4_Cl and (NH_4_)_2_SO_4_ did not yield sufficient results.

#### Determination of the optimal concentration of yeast extract for BC production

The effects of different yeast extract concentrations on cellulose production by *Rhizobium sp.* are shown in Fig. 2D. It was found that 2.17 g/L of cellulose was produced when yeast extract was added to the media at a concentration of 0.05%. An increase in cellulose productivity was observed when the concentration of yeast extract was increased by 0.1%, and reached 2.22 g/L when the concentration of 0.15% reached 2.27 g/L, and decreased when the concentration reached 0.2% reached 2.10 g/L.

### Environmental conditions

#### pH value

**Figure 3:**
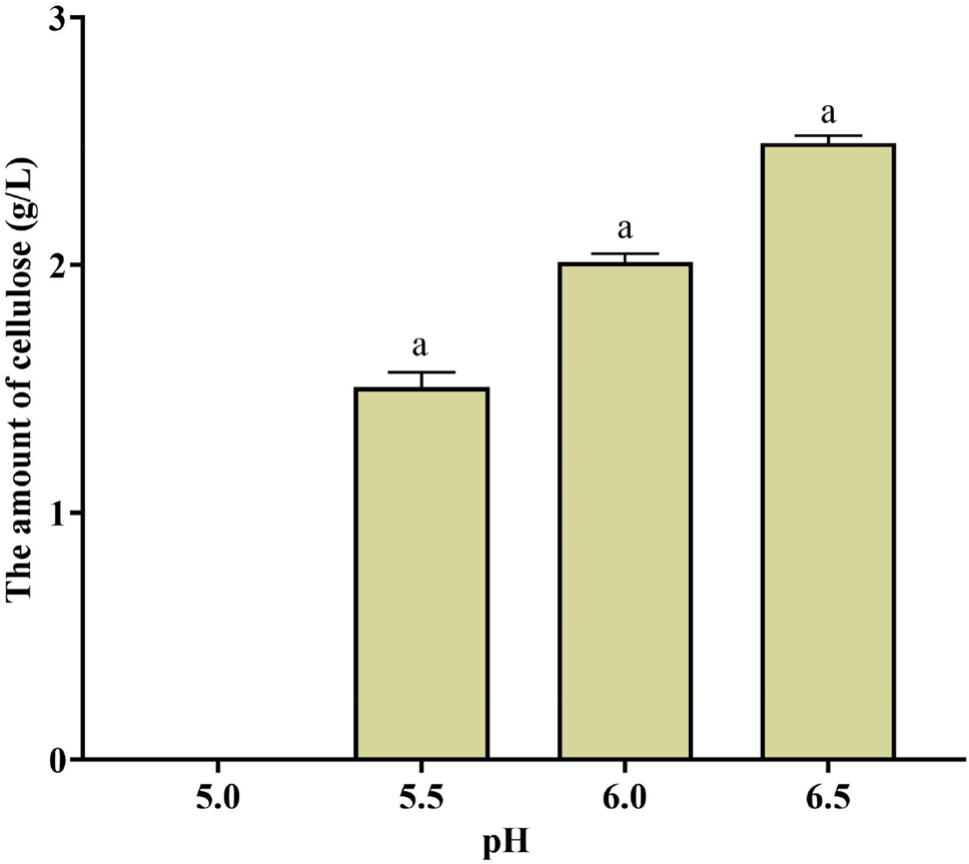
Effect of pH on cellulose production with glucose concentration (1.5%) and yeast extract concentration (0.15%) in static culture. The letter indicates significant differences P < 0.05 when comparing different treatments.

pH is an important factor in the control of the oxidative fermentation process for BC production. An acidic or near-neutral pH environment is considered to be optimal for the production of BC^70^. Figure 3 illustrates the impact of pH on cellulose production by *Rhizobium sp.*, employing a carbon source (glucose) at a concentration of 1.5% and a nitrogen source (yeast extract) at 0.15%. The manipulation of pH from 5.5 to 6.5 positively influenced bacterial cellulose production, with the optimal outcome achieved at pH 6.5, yielding 2.5 g/L.

#### Temperature

Temperature stands out as a crucial factor capable of shaping an organism's adaptation strategies for survival by impacting its regular homeostatic functions^70^. The optimum temperature of 30 °C was used to obtain the maximum yield of cellulose produced by *Rhizobium* sp. The amounts of cellulose produced were 2.21 g/L at 30 °C and 1.65 g/L at 35 °C. Temperature is a crucial variable affecting the growth and production of cellulose. Extensive research has shown that the ideal temperature range for cellulose production is 25–30 °C^64^. Several studies have shown that the maximum cellulose yield ranges from 28 to 30 °C^71^.

#### Aeration

The amount of cellulose produced in the flask covered with cotton was 2.5 g/L, whereas that produced in the uncovered flask was 2.03 g/L. The aerated cultures were examined to determine the effects of carbon dioxide and oxygen pressure. A previous study showed that the rate of cellulose production did not depend on the increase in the pressure of the oxygen. However, the production rate was dependent on the oxygen transfer rate, which decreased with an increase in the viscosity of the broth. This reduction in rate was attributed to the high carbon dioxide pressure. However, this decrease was reversed by a constant increase in the rate of airflow^72^.

### Characterization of BC/K.h, BC/K.h-Nis, BC/Rhiz, and BC/Rhiz-Nis

#### The thickness of BC/K.h, BC/K.h-Nis, BC/Rhiz, and BC/Rhiz-Nis

**Figure 4:**
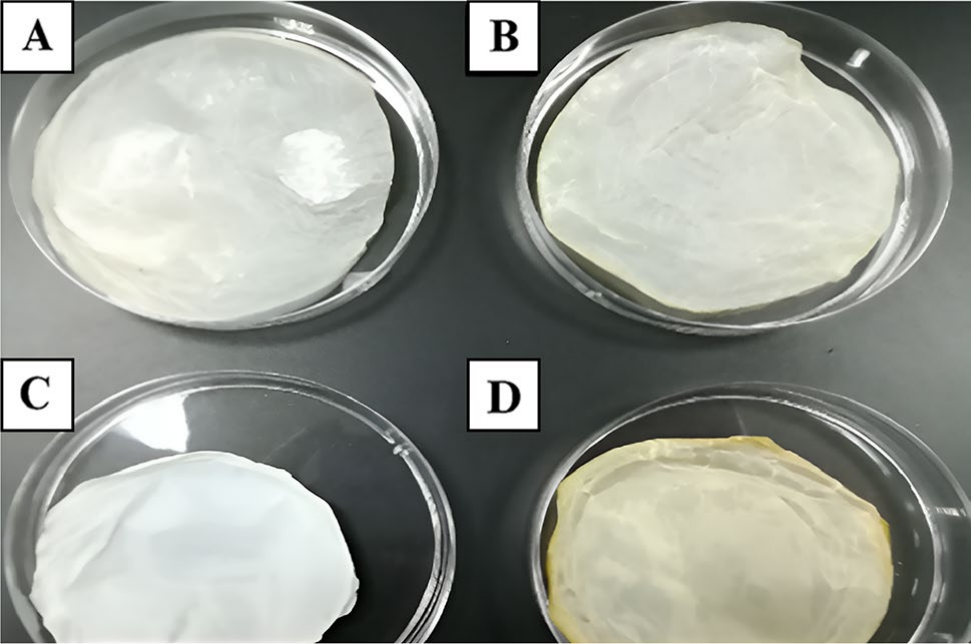
BC/K.h (**A**), BC/K.h-Nis (**B**), BC/Rhiz (**C**), and BC/Rhiz-Nis (**D**) membranes after drying for 24 h.

The thickness of the pure BC/K.h membrane measured 35 µm when wet and 10 µm when dry. In the wet state, the BC/K.h-Nis membrane exhibited a thickness of 42 µm, reducing to 13 µm when dry. The pure BC/Rhiz membrane's wet state thickness was 3 mm, aligning with the 10 µm thickness observed in dry conditions. The BC/Rhiz-Nis membrane displayed a wet state thickness of 34 µm, diminishing to 11 µm when dry. A visual representation of the results and sample details for the BC composite membranes can be found in Fig. 4 and Table 2, respectively.

**Table 2:** Characterization of BC/K.h, BC/K.h-Nis and BC/Rhiz and BC/Rhiz-Nis membranes.

Characterization	Unit	Material type			
		BC/K.h	BC/K.h-Nis	BC/Rhiz	BC/Rhiz-Nis
Porosity	%	60.5 ± 2	54.4 ± 2	57.8 ± 2	50.3 ± 2
Filtration	mL/min	24.18 ± 1.2	18.4 ± 1.2	22.33 ± 1.2	17.58 ± 1.2
Swelling	%	69 ± 4	55.1 ± 4	45.8 ± 4	35.6 ± 4
Thickness	µm	10 ± 0.1	13 ± 0.1	10 ± 0.1	11 ± 0.1

BC produced by *K. hansenii* (BC/K.h), BC composite with nisin (BC/K.h-Nis), BC produced by *Rhizobium* sp. (BC/Rhiz), BC composite with nisin (BC/Rhiz-Nis).

#### FE-SEM analysis of BC/K.h, BC/K.h-Nis, BC/Rhiz, and BC/Rhiz-Nis membranes

**Figure 5:**
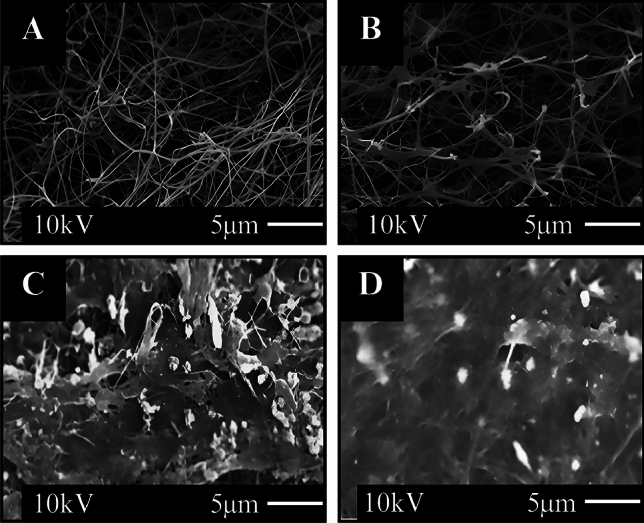
Structural analyses of freeze-dried samples of (**A**) BC/K.h, (**B**) BC/Rhiz, (**C**) BC/K.h-Nis, and (**D**) BC/Rhiz-Nis membranes.

FE-SEM was used to analyze the structure of lyophilized samples of BC/K.h, BC/K.h-Nis, BC/Rhiz, and BC/Rhiz-Nis membranes. The surfaces of the lyophilized samples were examined for comparative analysis. The results are shown in Fig. 5. The cellulose microfibrils in pure BC/K.h were randomly distributed and contained many voids, as shown in Fig. 5A.

#### FTIR analysis of BC/K.h, BC/K.h-Nis, BC/Rhiz, and BC/Rhiz-Nis membranes

**Figure 6:**
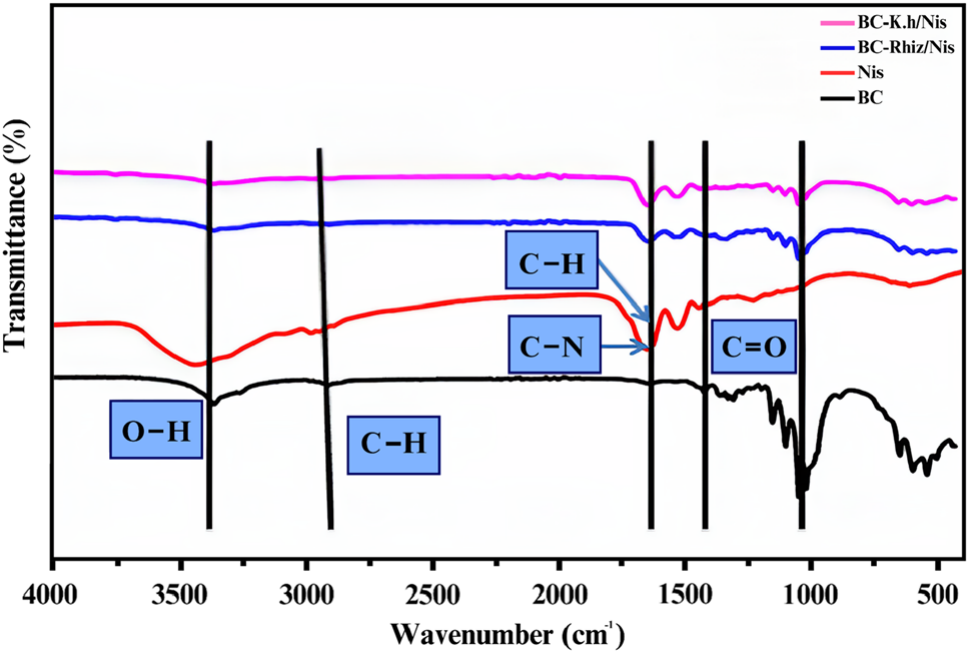
Fourier Transmission Infra-red Spectroscopy (FTIR) of BC/K.h, BC/K.h-Nis, BC/Rhiz, and BC/Rhiz-Nis membranes.

In this study, FTIR analyzes BC/K.h, BC/K.h-Nis, BC/Rhiz and BC/Rhiz-Nis membranes were performed to verify the synthesis of BC/K.h, BC/K.h-Nis, BC/Rhiz, and BC/Rhiz-Nis composites. The FTIR spectra of the nanocomposites of BC, nisin, and BC/Nis are shown in Fig. 6. The -OH functional group showed characteristic peaks at 3372/cm in the FTIR spectra of pure BC. Similarly, peaks corresponding to C–H stretching vibrations were observed at 2900 and 1620/cm. Moreover, the characteristic peaks for the C=O group were observed at 1420 and 1100/cm. As previously reported^73,74^, significant absorption bands associated with hydroxyl and amino acid functional groups were also observed for pure nisin.

In the 3302/cm region, vibrations originated from the O–H bond of pure nisin. In the FTIR spectra, the band corresponding to the absorption of the carbonyl (C=O) stretching of the secondary amide (amide I band) was detected at 1600.5/cm. In addition, peaks for the bending vibrations of the (C=O) were obtained at 1500.3/cm. Characteristics of BC/K.h-Nis composite clearly shows that the absorption peak for the -OH bending vibration occurs at 1028/cm for cellulose due to the presence of water molecules. The absorption peaks of nisin (amide II and N–H bending vibrations) appeared at 1642 and 1588/cm^75^. Owing to the dehydration of BC, the peak disappeared at 3424/cm. These results indicate that nisin interacts well with BC. Similar spectra were obtained for the BC/Rhiz-Nis.

#### WHC and WRR of BC/K.h, BC/K.h-Nis, BC/Rhiz, and BC/Rhiz-Nis membranes

For biomedical applications of BC, WHC and WRR are essential properties. WHC and WRR are directly related to changes in the BC matrix's surface area and pore size. The porous and fibrous nature of BC is responsible for its high WHC. According to^76^, hydrogen bonds hold water in pores and cellulose fibrils.

The pore sizes of the BC composites (BC/K.h-Nis and BC/Rhiz-Nis) were smaller than those of pure BC, as shown by the WHC and WRR results of this study. WHC of BC/K.h, BC/K.h-Nis, BC/Rhiz and BC/Rhiz-Nis were 115, 120, 130, and 126 times their dry weights, respectively.

**Figure 7:**
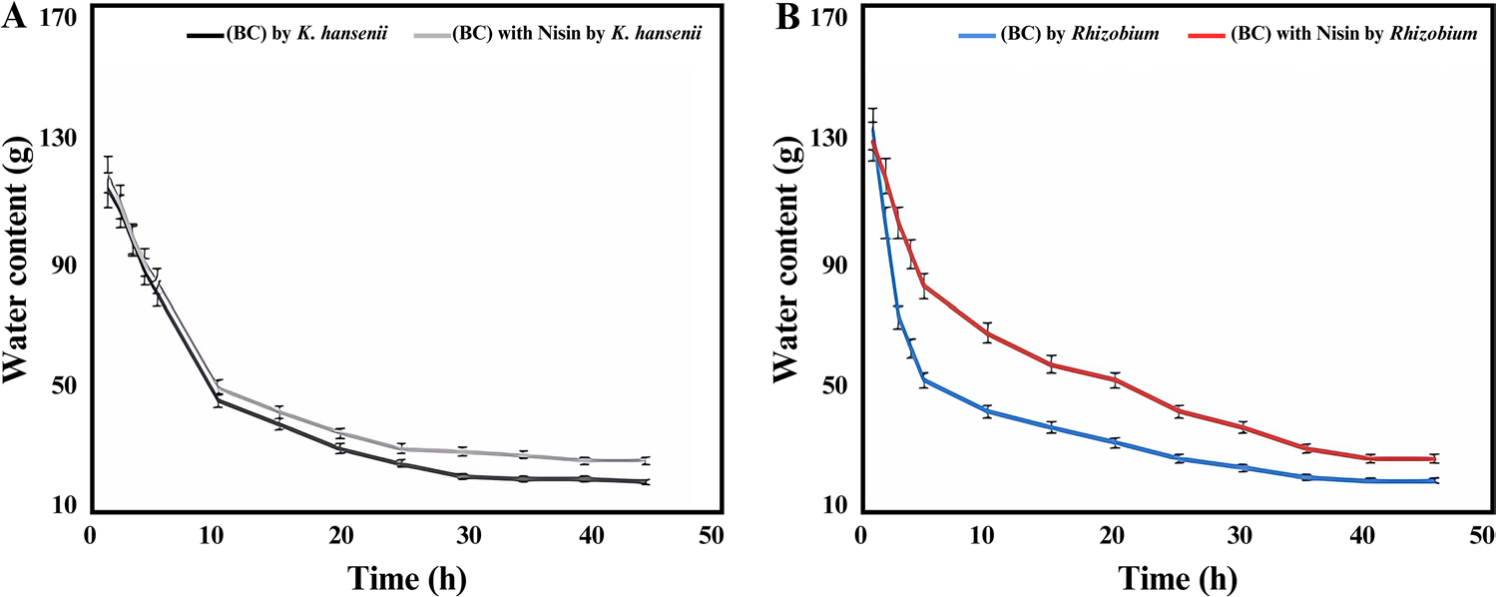
The WHC analyzes BC/K.h, BC/K.h-Nis (**A**), BC/Rhiz, BC/Rhiz-Nis (**B**) membranes.

The difference in pore size contributed to the increased WHC rates of the two composites compared to pure BC. As shown in Fig. 7A and B, the WHC of the composites was higher than that of the pure BC. These composites have smaller pore sizes, which lead to enhanced binding of the penetrating water molecules between the microfibrils. This led to a longer residence time for the water molecules in the BC fibrils of the composites. In comparison, the WRR obtained using pure BC is higher. The more space in the BC matrix, the more water can penetrate the material; however, water evaporates faster. Therefore, a larger pore size leads to higher WRR in BC samples^77^. Another study showed that changes in the physical properties of BC, such as WHC and WRR, were associated with changes in various parameters, such as microfibril arrangement, pore size, pore volume, and surface area following structural modifications^78^.

In summary, pore size, fibril arrangement, and pore volume have an influence on the WHC and WRR. In addition, modifying pure BC with nisin resulted in the restructuring of BC fibrils, leading to an improved water absorption capacity.

#### The porosity analysis of BC/K.h, BC/K.h-Nis, BC/Rhiz, and BC/Rhiz-Nis membranes

**Figure 8:**
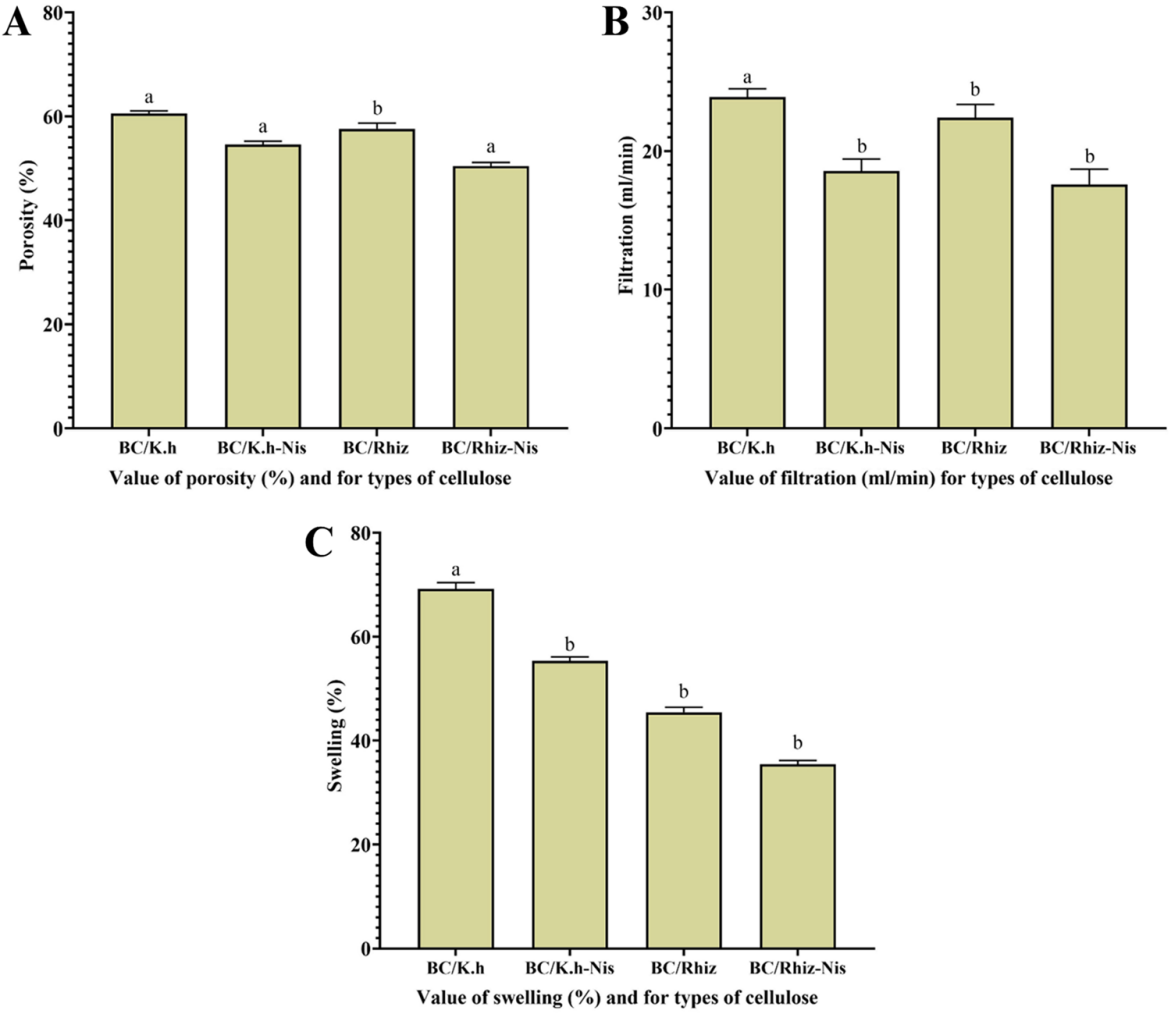
The porosity, filtration, and swelling analysis of BC/K.h, BC/K.h-Nis, BC/Rhiz, and BC/Rhiz-Nis membranes. Letters indicate significant differences P < 0.05 when comparing different treatments.

The porosity of the membrane is quantified as the fraction of the void volume within the membrane, which is precisely calculated by dividing the volume occupied by the pores by the total volume enclosed by the membrane^79^. Porosity of BC/K.h, BC/K.h-Nis, BC/Rhiz and BC/Rhiz-Nis were 60.5%, 54.4%, 57.8%, and 50.3%, respectively. The results are shown in Fig. 8A. Our experimental results are consistent with the findings of dos Santos et al.^80^; when examining the X-ray microtomography parameters of BC and BC-Nis membranes, it was observed that BC membranes exhibited greater connectivity compared to the BC-Nis membranes. Furthermore, the inclusion of nisin resulted in a 2% reduction in membrane porosity, which aligns with findings from SEM images indicating interconnected pores distributed evenly throughout the sample^80^.

#### Filtration analyses of BC/K.h, BC/K.h-Nis, BC/Rhiz, and BC/Rhiz-Nis membranes

A filtration study was performed to investigate the ability of the fluids to permeate the composites. Figure 8B shows the water permeation properties of the composite membranes. The results of filtration analyses for BC/K.h, BC/K.h-Nis, BC/Rhiz and BC/Rhiz-Nis revealed 24.18 mL/min, 18.4 mL/min, 22.33 mL/min, and 17.58 mL/min, respectively. Filtration capacity of BC/K.h-Nis, BC/Rhiz, and BC/Rhiz-Nis composite membranes were lower than BC/K.h. This can be attributed to the different physical properties of the composite membranes. The fibrous network of BC/K.h was spatially better arranged than those of the other composites (Fig. 5A). For example, the filtration capacity of BC/K.h-Nis is much lower than that of the other membranes. This is probably due to the tightly woven fiber network, which significantly reduces the filtration of the liquid yield compared to the other composites (Fig. 5B). However, similar structural properties are evident in the close numerical relationship between BC/K.h and BC/Rhiz compositions. Owing to their high hydrophilicity, BC/K.h and BC/Rhiz had higher filtration rates than BC/Rhiz-Nis and BC/K.h-Nis. Water molecules were selectively dissolved in the non-composites (BC/K.h and BC/Rhiz) and diffused mainly through the BC layer, resulting in higher filtration rates.

Filtration capacities of BC/K.h, BC/K.h-Nis, BC/Rhiz and BC/Rhiz-Nis were determined, and the results are shown below:

- In the case of pure BC/K.h, the filtration time for 100 mL of distilled water was 4.01 min, resulting in a residual water volume of 97 mL.
- The BC/K.h-Nis membrane required 5 min to filter 100 mL of distilled water, leaving a residual water volume of 92 mL.
- For the BC/Rhiz membrane, the filtration time for 100 mL of distilled water was 4.3 min, with a residual water volume of 96 mL.
- The BC/Rhiz-Nis membrane demonstrated a filtration time of 4.7 min for 100 mL of distilled water, and the residual water volume after filtration was 92 mL.

Filtration is an essential and routinely used technique in practice. The objective or application typically determines the pore size of the filter. These include mycoplasma removal, laboratory sterilization, media filtration, biological fluids, gases, solutions, and solvents^81,82^.

#### Swelling analysis of BC/K.h, BC/K.h-Nis, BC/Rhiz, and BC/Rhiz-Nis membranes

The swelling ability of bacterial cellulose refers to the ability of the cellulose to absorb and hold onto water or other liquids. Bacterial cellulose typically has a high swelling ability due to its porous structure and hydrophilic nature^83^. Swelling abilities of BC/K.h, BC/K.h-Nis, BC/Rhiz and BC/Rhiz-Nis were investigated to determine the ability of water to penetrate the structure (Fig. 8C). Swelling results for BC/K.h, BC/K.h-Nis, BC/Rhiz and BC/Rhiz-Nis were 69%, 55.1%, 45.8%, and 35.6%, respectively. The results are presented in Table 2. According to^84^, swelling and crystalline linearity are directly proportional to the hydrophilicity/hydrophobicity of the material.

According to^84,85^, owing to the enormous hydrophilic properties of BC, water can easily penetrate cellulose and break the van der Waals force between the molecules, resulting in hydrogen bonds. The results showed that the swelling value of the *K. hansenii* cellulose product was higher than those of the other composites (BC/K.h-Nis, BC/Rhiz, and BC/Rhiz-Nis) due to its chemical and physical properties. Chemically, pure BC swells more than BC/Nis and BC/Rhiz because it is naturally hydrophilic and can absorb large amounts of water. Physically, BC has a highly porous fiber network with many interspersed and perforated voids. The BC composite (BC/K.h-Nis, BC/Rhiz, and BC/Rhiz-Nis) may have affected the physical properties of BC/K.h such that this fibrous network is impaired/reduced, and swelling values are ultimately lower than pure BC.

Swelling, which is influenced by several factors, is essential for understanding hydrogel structures. In the case of cellulose, swelling maintains the reticulated structure as part of particles, fibers, or as a film. However, the physical properties changed significantly, and the sample volume increased due to the swelling agent's absorption. However, the swelling of cellulose has a unique property: destruction of the supramolecular structure of cellulose. Thus, both processes were controlled using the same solvent and biopolymer. Therefore, depending on the properties of cellulose and operating conditions, there is no clear boundary between the swelling process and the same system^86^.

#### Antibacterial activity of BC/K.h, BC/K.h-Nis, BC/Rhiz and BC/Rhiz-Nis determined by the disk diffusion method

**Figure 9:**
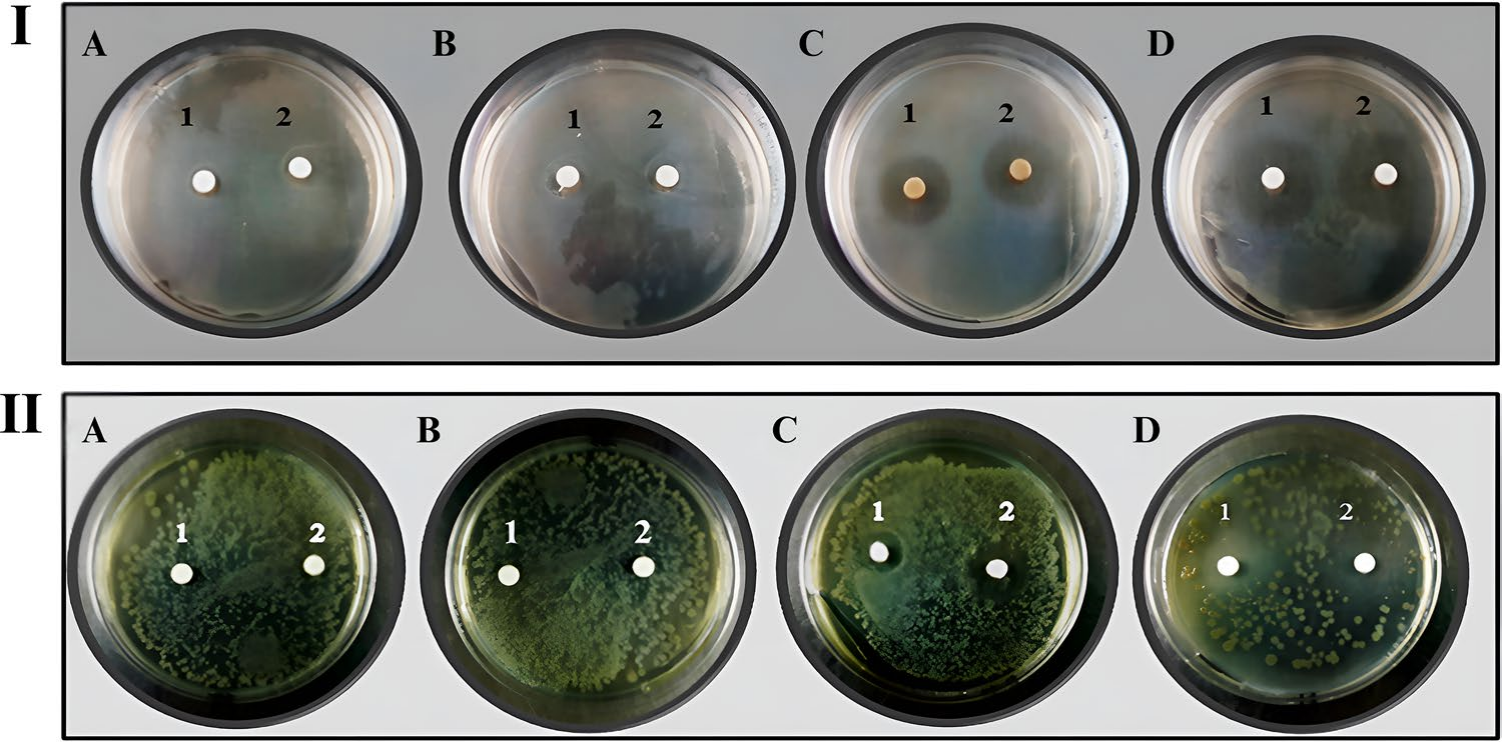
The inhibition zone of *E. coli* (I) and *P. aeruginosa* (II). (**A-1**) Cellulose discs produced from *K. hansenii* were treated with 100 IU nisin. (**A-2**) Cellulose discs produced from *Rhizobium* sp. treated with 100 IU nisin. (**B-1**) Cellulose discs produced from *K. hansenii* were treated with 200 IU nisin. (**B-2**) Cellulose discs produced from *Rhizobium* sp. were treated with 200 IU nisin. (**C-1**) Cellulose discs produced from *K. hansenii* were treated with 300 IU nisin. (**C-2**) Cellulose discs produced from *Rhizobium* sp. were treated with nisin at a concentration of 300 IU. (**D-1**) Cellulose discs produced from *K. hansenii* were treated with 400 IU nisin. (**D-2**) Cellulose discs produced from *Rhizobium* sp. were treated with 400 IU nisin.

This study investigated the efficacy of *K. hansenii* and *Rhizobium sp.* inhibitory cellulose disks impregnated with different concentrations of nisin against *P. aeruginosa* and *E. coli* for 24 h. The cellulose disks produced by *K. hansenii* and *Rhizobium sp.*, and impregnated with nisin (400 IU), were effectively inhibited (Fig. 9). The cellulose disks of *Rhizobium sp.* had a total diameter of (23 mm) inhibition zone for *E. coli*, and (20.1 mm) inhibition zone for *P. aeruginosa*.

**Figure 10:**
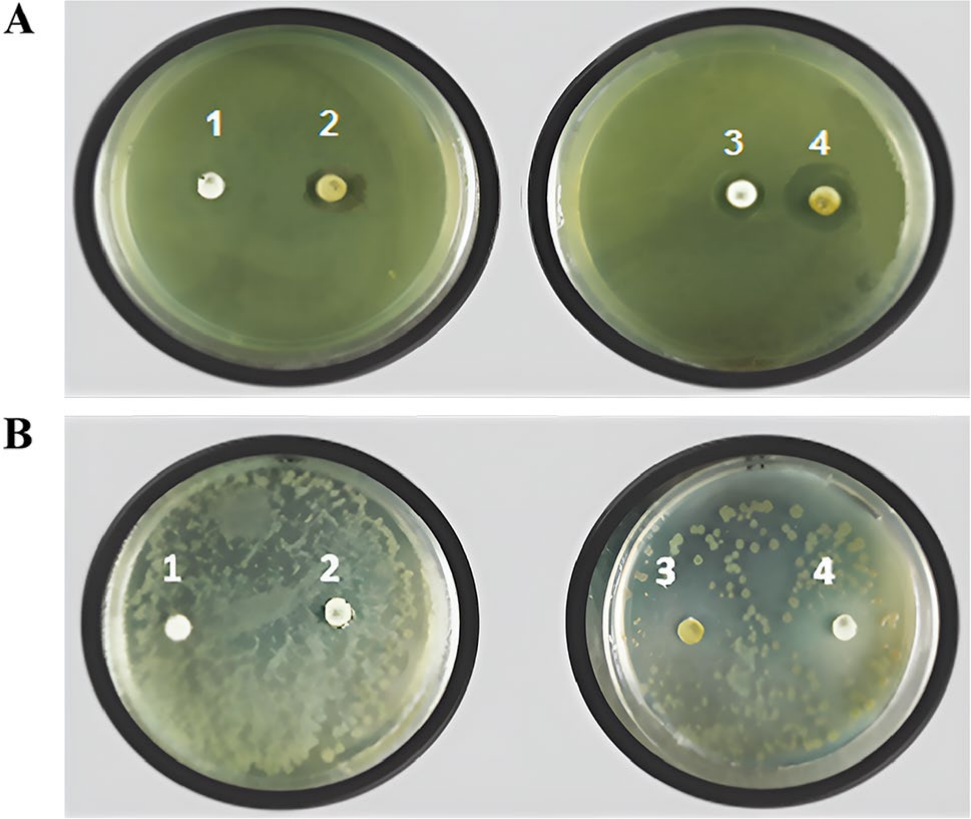
The inhibition zone of *E.coli* (**A**) and *P. aeruginosa* (**B**) bacteria by commercial antibiotics discs: (**1**) Penicillin, (**2**) Ampicillin, (**3**) Tetracycline, and (**4**) Chloramphenicol.

Cellulose disks produced by *K. hansenii* had a diameter of 21.5 mm for *E. coli* and 19.3 mm for *P. aeruginosa*. The results confirmed the ability of the formulation to inhibit both negative and positive Gram staining assays and nisin’s ability to overlap the fibers’ cellulose membranes. These results were consistent with their ability to inhibit *P. aeruginosa*^87^ and *E. coli*^88^. Antibiotic disks against *E. coli* (A) and *P. aeruginosa* (B) were used as controls for comparison with experimental disks (Fig. 10).

## Conclusion

The remarkable versatility of BC as a biomaterial has led to its extensive use in developing high-quality paper, nanocomposites, wound dressings, and artificial vascular constructs. In this study, a novel BC producer, *Rhizobium sp*., was isolated from bean roots and evaluated for its ability to produce BC in simple, cost-effective media. In this study, the growth and BC production conditions were optimized for *Rhizobium* sp., and the BC produced by *Rhizobium sp*. was successfully modified with nisin, which exhibits broad-spectrum inhibitory activity against various microorganisms. The resulting BC was compared with the BC produced by *K. hansenii*. The results showed that BC produced by *Rhizobium sp*. had properties similar to those of BC produced by *K. hansenii*.

Moreover, BC-Nis composites showed remarkable inhibitory activity against *E. coli* and *P. aeruginosa*. These results suggest that BC production by *Rhizobium sp*. and its modification with nisin could be used in various industries. To improve BC production and shorten the production time, further molecular studies are required to isolate the genes responsible for BC production in *Rhizobium sp*. and improve their expression. In addition, the potential use of BC composites produced by *Rhizobium sp*. and other materials should be investigated for various applications in bioelectronics, medicine, agriculture, and the food industry. Overall, this study provides valuable insights into the production and modification of BC by *Rhizobium sp*. and its potential applications in various fields.

## Data Availability

The data used to support the findings of this study are included within the article.

## Abbreviations

3D: Three dimensional
BC: Bacterial cellulose
CR: Congo Red
FE-SEM: Field-emission scanning electron microscopy
FTIR: Fourier transform infrared spectroscopy
H_2_S: Hydrogen sulfide
HS: Hestrin-Schramm media
MYA: Mannitol yeast extract agar
SIM: Sulfur, indole, motility
WHC: Water-holding capacity
WRR: Water release rate
